# Antibacterial-Coated Suture in Reducing Surgical Site Infection in Breast Surgery: A Prospective Study

**DOI:** 10.1155/2012/819578

**Published:** 2012-12-19

**Authors:** Enora Laas, Cécile Poilroux, Corinne Bézu, Charles Coutant, Serge Uzan, Roman Rouzier, Elisabeth Chéreau

**Affiliations:** Service de Gynécologie-Obtétrique, Hôpital Tenon, Assistance Publique des Hôpitaux de Paris, CancerEst, Université Pierre et Marie Curie-Paris 6 75020 Paris, France

## Abstract

*Background*. To reduce the incidence of microbial colonization of suture material, Triclosan- (TC-)coated suture materials have been developed. The aim of this study was to assess the incidence of suture-related complications (SRC) in breast surgery with and without the use of TC-coated sutures. *Methods*. We performed a study on two consecutive periods: 92 patients underwent breast surgery with conventional sutures (Group 1) and 98 with TC-coated sutures (Group 2). We performed subgroups analyses and developed a model to predict SRC in Group 1 and tested its clinical efficacy in Group 2 using a nomogram-based approach. *Results*. The SRC rates were 13% in Group 1 and 8% in Group 2. We found that some subgroups may benefit from TC-coated sutures. The discrimination obtained from a logistic regression model developed in Group 1 and based on multifocality, age and axillary lymphadenectomy was 0.88 (95% CI 0.77–0.95) (*P* < 10^−4^). There was a significant difference in Group 2 between predicted probabilities and observed percentages (*P* < 10^−5^). The predicted and observed proportions of complications in the high-risk group were 38% and 13%, respectively. *Conclusion*. This study used individual predictions of SRC and showed that using TC-coated suture may prevent SRC. This was particularly significant in high-risk patients.

## 1. Introduction

Most of the patients diagnosed with breast cancer will undergo surgery, with a significant risk of complications. These complications, although they are often minor, affect 30% of all patients and entail such adverse effects as impaired quality of life, delays in adjuvant therapy, higher costs, and increased duration of hospitalization.

The most common adverse effects are hematoma (2–10%), seroma (10–85%), and surgical site infection (SSI) (0.8–45.8%) [1, 2]. The measures used to prevent these nosocomial infections include prophylactic antibiotic treatment, preoperative preparation of the patient, and respect for the rules of asepsis and postoperative care protocols. Most of these measures have been formally evaluated and proven to be valuable; however, there is still a need for improved prevention of surgical complications, especially those involving infectious matters [3–5].

Suture material is known to be a potential agent of infection [6–9]. To prevent microbial colonization of suture material in operative wounds, Triclosan-coated polyglactin 910 suture materials with antibacterial activity (Vicryl Plus and Monocryl Plus Ethicon GmbH, Nordersdedt, Germany) have been developed. Triclosan (TC) is a broad-spectrum phenol family antiseptic, used for more than 30 years as a safe and effective antimicrobial agent [10], against the most common pathogen agents that cause SSI: *S. aureus* and *S. epidermidis*. The antimicrobial efficacy of this material in reducing both bacterial adherence to the suture and microbial viability have been proven in vitro [11, 12] and in animal models [13–15].

Coated sutures with TC were compared clinically to nonimpregnated suture material in extragynecological surgery, and were shown to perform as well or better than traditional sutures with respect to intraoperative handling and wound healing in pediatric general surgery [16], pediatric neurosurgery [17], thoracic [18], and abdominal surgery [19, 20]. However, other studies suggest that TC-coated sutures could be inefficient or might have potential adverse effects as wound dehiscence, and should be used with caution [21, 22]. Recently, two studies have tried to use TC-coated suture in breast surgery, and showed better cosmetic outcomes and efficiency in reducing SSI [23, 24].

The aim of the current study was to assess the incidence of both suture-related complications (SRC) and surgical site infections (SSI) between two populations: those with and without the use of Triclosan-coated Vicryl Plus.

## 2. Materials and Methods

We analysed prospectively collected data in two identical summer periods at Tenon Hospital, Paris, France. During the first period, from June to August 2009, 92 patients underwent breast surgery with conventional sutures (Group 1). From June to August 2010, 98 patients underwent breast surgery with TC-coated sutures (Group 2).

All the women gave informed written consent to the therapeutic procedures and to the analysis of data related to their pathology. The protocol was approved by Ethics Committee.

Breast interventions were performed for both malignant and nonmalignant tumors. Patients who underwent reconstructive surgery were excluded. Details regarding patient characteristics including Body Mass Index (BMI), National Nosocomial Infections Surveillance (NNIS) status [25], diabetes mellitus status, and tobacco consumption were collected prospectively. Complementary data obtained from patient files included patient sociodemographic characteristics, tumoral characteristics, peri- and postoperative data. Intervention details and postoperative complications were also collected prospectively, during hospitalization. Patients were followed up at 15 and 30 days after discharge but could have been seen in an emergency. We recorded SRC, which included SSI (including fever, wound discharge, and surgical site infection), and ‘‘cutaneous complications” (including delay to wound healing and wound dehiscence, allergy, and necrosis).

SSI within the 30 first days after surgery was considered to be related to surgery and was classified in terms of severity of the infection, according to the French recommendations for nosocomial infections [26].

### 2.1. Surgical Procedure

All patients underwent preoperative chlorexidine skin cleansing and skin preparation with double chlorexidine washing. During the first period (Group 1), we used classical sutures (Vicryl and Monocryl, Ethicon), and during the second period (Group 2) we used sutures coated with TC (Vicryl Plus and Monocryl Plus, Ethicon).

We used drainage at the site of mastectomy and on the axillary basin. Other drainage materials could be placed in case of a large lumpectomy. Prophylactic antibiotic treatment was performed according to the anesthesiology unit protocol: no systematic treatment in case of conservative surgery or cefazoline 2 g IV during mastectomy or axillary lymphadenectomy. Clindamycine and gentamicine were used in case of allergy. Postoperative anticoagulation was performed according to the recommendations of the French society of anesthesiology [27].

### 2.2. Statistical Analysis

The endpoint was to compare SRC between two populations, with and without TC-coated sutures. We used prediction of expected SRC among the TC-treated population, based on the characteristics of this population.

Qualitative variables were compared with the chi-square test or Fisher's exact test. A *t*-test was used for continuous variables, if normality could be assumed. All reported tests were two-sided (*P* ≤ 0.05 was considered significant).

We performed subgroups analyses in patients with known risk factors of infection to determine if some patients may benefit from TC-coated sutures.

All significant variables in the univariate analysis were subjected to logistic regression, as were factors used in the literature (age, diabetes, and BMI) to assess independent predictors of breast surgery complications. Akaike Information Criteria (AIC) was used to perform backward stepwise variable selection among Group 1. The model predictive ability was validated with a 1000-bootstrap replicate. Individual probabilities of complications were calculated with this model.

Discrimination was quantified with the area under the receiver operating characteristic curve (AUC). Confidence intervals were calculated using a bias-corrected bootstrap with 1000 iterations.

Calibration corresponds to the agreement between the observed outcome frequencies and the predicted probabilities. The results are displayed in a calibration graph that shows the relationship between the observed outcome frequencies and the predicted probabilities for two groups of patients categorized according to a median split (semicohorts with the lowest/highest predicted complication rate). Well-calibrated models have *a* = 0 and *b* = 1. Therefore, a sensible measure of calibration (the unreliability index) is a likelihood ratio statistic testing the null hypothesis that *a* = 0 and *b* = 1.

To evaluate the efficiency of TC-coated sutures, we compared individual predicted probabilities of complications using the model versus observed complication rate in Group 2 [28]. The treatment was considered efficient if observed probabilities were lower than predicted, not efficient if they were equal, and harmful if the observed rate of complications was greater than the predicted rate.

## 3. Results

During these two periods, 190 patients underwent breast surgery. Among them, wound closure was performed with traditional suture material in 92 (Group 1) and with TC-coated sutures in 98 (Group 2).

Patients were similar in both groups with respect to sociodemographic and tumoral characteristics (Table 1). There were no significant differences for the type of surgery, the rate of drainage placement and duration, and the antibiotic prophylactic treatment. NNIS score repartition did not differ between the groups. Patients in Group 2 had a higher median operative duration (80 min versus 60 min, *P* < 0.001).

The SRC rates were 13% (12/92) in Group 1 and 8% (8/98) in Group 2. This difference was not significantly different (*P* = 0.3) (Table 2). SSI occurred in 10 patients with classical sutures (11%) and in 6 patients whose wounds were closed by TC-coated sutures (6%) (*P* = 0.2).

**Figure 1:**
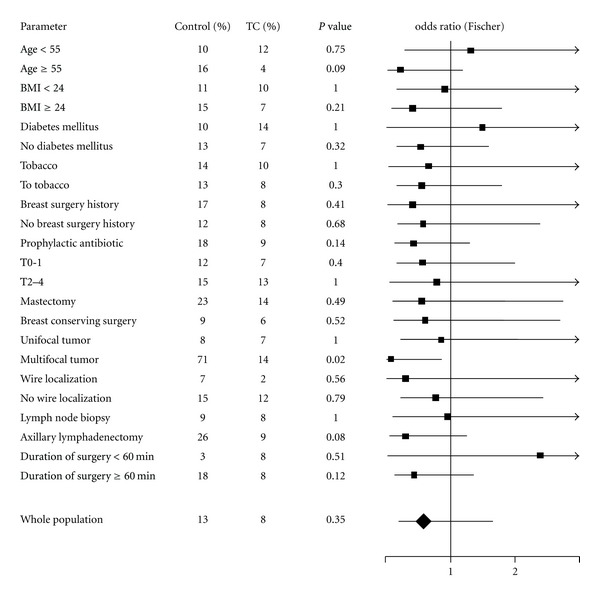
Forest plot representing odds ratios and their confidence intervals for subgroups.

We investigated sources of heterogeneity by subgroup analyses. Results are reported in Figure 1 as a forest plot representing odds ratios and their confidence intervals for subgroups. Almost all odds ratios were below 1. However, TC-coated sutures had a significant protective effect against SRC only in multifocal tumors. The protective effect fell short of reaching statistical significance in case of age >55 axillary dissection and duration of surgery >60 min. This latter analysis suggest that a model-based approach may help selecting patient for a benefit of TC-coated sutures.

In univariate analysis, significant risk factors for SRC in Group 1 were conservative surgery, axillary lymphadenectomy, axillary drainage, breast drainage duration, postoperative anticoagulation, and multifocality. Based on a multivariate analysis, we developed a model to predict individual risk of SRC, using variables and interaction, which increase Akaike Information Criteria (axillary lymphadenectomy and age in our model). Variables selected in the model are presented in Table 3.

**Figure 2:**
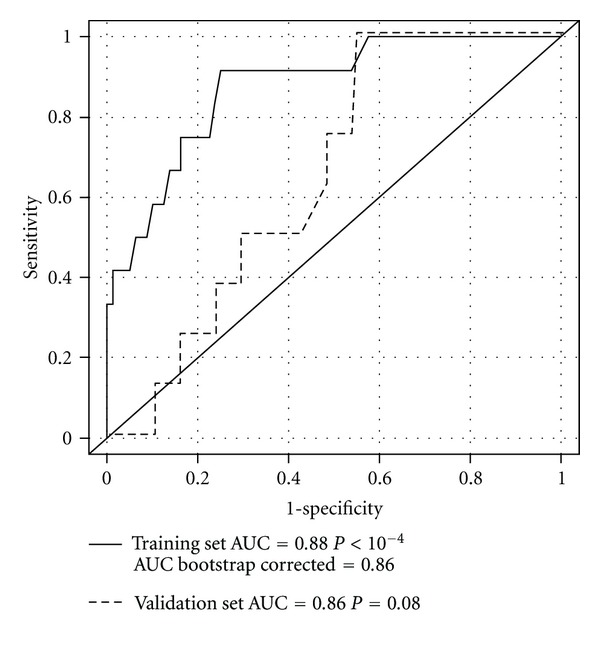
Receiver-operating characteristic curve of predictions from the model to predict complications in the training set (traditional suture) (plain curve) and in the validation set (TC-coated suture) (dotted curve).

The AUC obtained with this logistic regression model in the training population was 0.88 (95% CI 0.77–0.95); after bootstrap validation, the AUC was 0.86 (*P* < 10^−4^) (Figure 2).

We calculated the individual probability of complication with a specific model built based on the data from Group 1.

**Figure 3:**
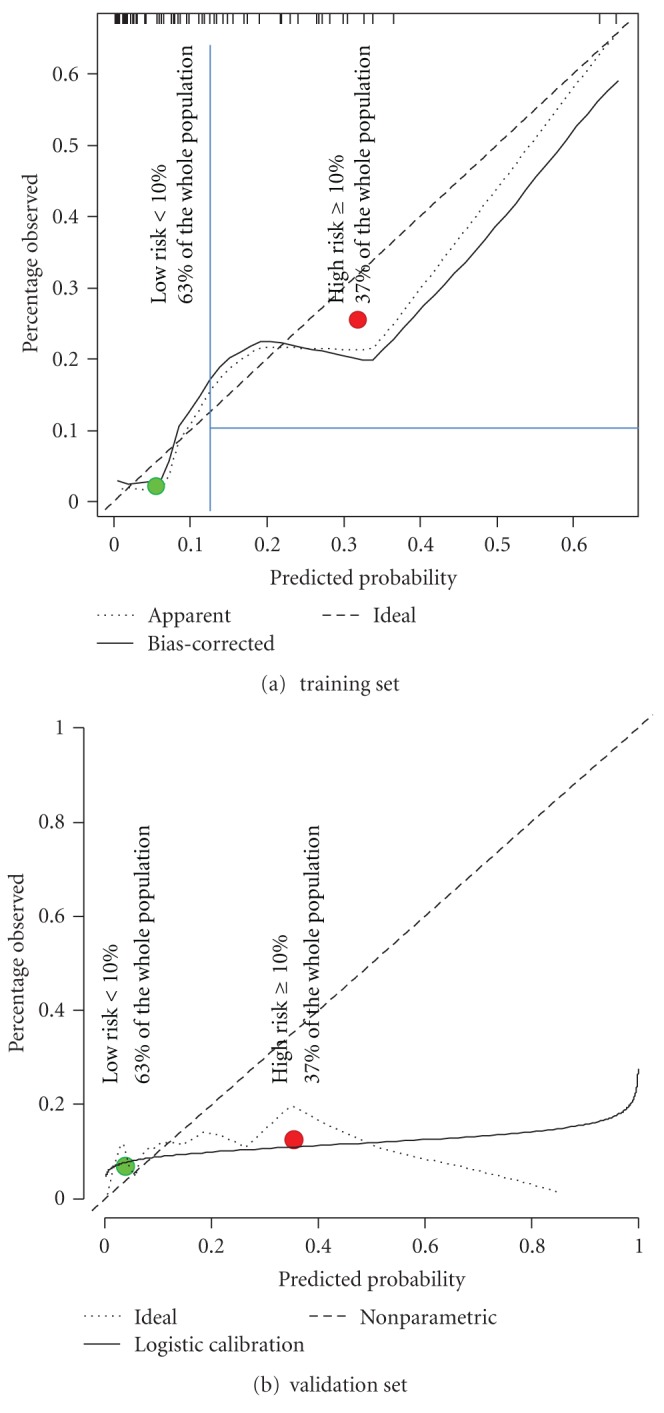
Calibration of the model to predict complications.

We used this model to predict the individual probability of SRC in Group 2. Overall, the observed rates of complication were not concordant with predictions in this subgroup, as demonstrated by discrimination and calibration. Discrimination assessed by AUC did not reach statistical significance (AUC = 0.65, *P* = 0.08) (Figure 2). The calibration curve was unsatisfactory (Figure 3): there was a significant difference between predicted probabilities and observed percentages.

In Figure 3, patients were separated in two subgroups according to their predicted probability of complications (*x*-axis): low risk for predicted risk <10% and high risk for predicted risk ≥10%. The observed complication rate is reported on the *y*-axis. Perfect predictions are plotted on the ideal line: intercept at (0, 0), slope: 45°. The predicted probability and observed proportion of complications among patients with the lowest risk of complications were 2.2% and 5.9%, respectively. The predicted probability and observed proportion of patients who experienced complications in the high-risk group were 38% and 13%, respectively (*P* < 10^−5^). Thus, data obtained using a model developed to predict the frequency of complications among women with traditional wound closures suggest that using TC-coated suture material decreased SRC. This was particularly clinically significant in high-risk patients.

We calculated sample size of a randomized trial to demonstrate the protective effect of TC-coated sutures. In the general population, the inclusion of 629 patients in each arm (alpha = 5%, power = 80%) would be necessary based on risks of SCR of 13% and 8% in the control arm and TC-coated arm, respectively. By selecting only patients with a risk of SCR more than 10% based on our model, the inclusion of only 55 patients in each arm is necessary (alpha = 5%, power = 80%), based on risks of SCR of 38% and 13% in the control arm and TC-coated arm, respectively.

## 4. Discussion

The primary objective of this prospective study was to evaluate the efficiency of an antibacterial-coated suture to decrease both SSI and other SRC (such as skin necroses, delay to wound healing, or wound dehiscence) after breast surgery.

SRC rates were 13% and 8%, respectively, for Group 1 and Group 2, which is consistent with most studies (0.8–45%) [29–31]. The observed complication rate for patients treated with TC-coated suture material seemed to be similar to that observed during the first study period (Table 2) and may be wrongly interpreted as unsatisfactory. However, based on patient and surgery characteristics, the complication rate was expected to be higher. This was shown through the use of a model that was based and validated on the classical population.

In the present study, the new treatment allocation was sutures coated with Triclosan. Triclosan is a broad-spectrum antimicrobial agent present in various topical products, (soaps, surgical scrubs, toothpaste …) for over 30 years [32]. It has antimicrobial activity against the most common organisms that cause SSI, including Methicillin-Resistant *Staphylococcus aureus* (MRSA), and Methicillin-Resistant *Staphylococcus epidermidis* [10, 13]. After resistance for *Pseudomonas aeruginosa* was found in vitro, Triclosan was suspected to induce resistance to antibiotic [33, 34]. However, these results were not confirmed for other bacteria [35] or in vivo [36, 37].

Triclosan can reduce the extent of bacterial colonization usually existing on suture material. Its efficiency in reducing the infection of surgical wounds has been reported in gynecological [23, 24] and extragynecologic surgery (neurosurgery, thoracic, and abdominal surgery) [17–19]. Ford et al. showed that intraoperative handling of coated polyglactin 910 sutures was indistinguishable from that with Triclosan [16]. However, the advantages of TC-coated sutures were criticized in several randomized trials. In a few series, the use of TC-coated sutures failed to reduce the rate of wound infection after appendectomy or head and neck surgery [20]. In a trial concerning breast reduction surgery, Deliaert et al. reported a higher wound dehiscence rate with TC-coated sutures than with conventional sutures [21]. It is important to note that the expected potential beneficial effects of TC-coated sutures extend beyond merely the prevention of infection. In 2007, Gómez-Alonso et al. demonstrated the efficacy of these sutures in preventing bacterial colonization and modulating the inflammatory response, which allowed better tissue healing [14].

The risk factors of suture-related complications found in our predictive model based on logistic regression are tumor multifocality, axillary lymphadenectomy, and age.

Comparing the effects of various suture materials in reducing SSI and poor wound healing in a randomized trial may require the inclusion of several hundred patients, because the prevalence of these complications is low. Models may be a useful way to test a new treatment or therapeutic strategy without randomization. They can provide theoretical individualized outcomes based on validated multivariate analyses [3]. Any difference between the predicted probability and the observed proportion reflects that the new treatment allocation or strategy modifies the outcome. Of course, such a multivariate analysis can never adjust for unmeasured or unknown confounders. The superiority of a randomized study is not denied but may require inclusion of several hundred patients with selection criteria that limit its generalizability [23]. We report in our study that selecting patients based on their baseline risk of SCR may help to decrease the number of patients to include in a randomized study by a factor 10. This is clearly a major advantage of our approach, which indeed prevents to unnecessarily include patients that will have no benefit of treatment because of their very low risk. The interest in a randomized trial is also driven by the additional cost of the new strategy. In our practice, we used six sutures: the involved cost was 20€96 with TC-coated sutures, as compared to 18€26 with classical sutures. Olsen et al. found that the attributable cost of SSI after breast surgery was $4,091 [29]. Therefore, any decrease in SRC will easily translate to medicoeconomic benefits.

There are some limitations to our study. First, the number of cases was limited (*n* = 190) so the interpretation of our results may lack statistical power. Second, the robustness of our predictor model has been validated in the same population. Notably, this was performed using a validated bootstrap method [38–41] for a large group of patients and provides multivariate stratification for maximizing the accuracy of estimation that might be superior to simple matching, especially if it is based on more extensive information (larger datasets). Third, the number of criteria that are included in the model is limited. Finally, the study was performed at a single center in France over two different time periods and using two different types of suture material. Using the same periods at a year's interval would prevent bias associated with season or residents.

In conclusion, in this preliminary study, age, axillary lymphadenectomy, and multifocal tumor were the major predisposing factors leading to breast wound infection and suture-related complications. TC-coated sutures seem to reduce the rate of complications after the surgical treatment of breast pathologies, particularly in the high-risk group.

## Figures and Tables

**Table 1:** Patient, tumor, and surgical characteristics.

	Group 1	Group 2	*P *
	*n* = 92 (%)	*n* = 98 (%)	
Patient characteristics			

Age med (min–max)	55.5 (14–86)	54.5 (23–87)	0.6
Breast surgery history	24 (26)	26 (26)	0.9
Radiotherapy history	1 (1)	2 (2)	0.6
Medical history			
Diabetes mellitus	10 (11)	7 (7)	0.4
Tobacco use	14 (15)	20 (20)	0.4
High blood pressure	28 (30)	25 (25)	0.4
BMI med (min–max)	23.9 (16.4–42.2)	24.8 (17.5–48)	0.8
Corticosteroid therapy	1 (1)	1 (1)	0.9
Immunodepression	1 (1)	1 (1)	0.9

Surgery			

Mastectomy	26 (28)	28 (28)	0.9
Conservative surgery	56 (61)	64 (65)	0.5
Revision lumpectomy	3 (3)	4 (4)	0.7
Lymph node biopsy	23 (25)	36 (37)	0.08
Axillary lymphadenectomy	34 (37)	33 (34)	0.6
Wire localization	27 (29)	41 (42)	0.07

Tumor characteristics			

Bilateral	7 (8)	14 (14)	0.14
Malignant tumor	65 (76)	72 (77)	0.9
Tumor size med (min–max)	20.8 (4–65)	21 (1–70)	0.8
Multifocality	7 (8)	14 (14)	0.14
Number of axillary lymph nodes med (min–max)	11 (4–23)	10 (5–22)	0.4

Neoadjuvant therapies			

Neoadjuvant chemotherapy	7 (11)	5 (7)	0.4

Group 1: traditional suture material.

Group 2: sutures coated with triclosan.

**Table 2:** Surgical and postoperative course.

	Group 1	Group 2	*P *
	*n* = 92 (%)	*n* = 98 (%)	
Surgical course			

Prophylactic antibiotic treatment	66 (72)	80 (82)	0.1
Operation duration med (min–max)	60 (20–220)	80 (20–220)	0.0003
NNIS score			
0	66 (88)	59 (74)	
1	9 (12)	20 (25)	0.07
2	0	1 (1)	
Postoperative anticoagulation	64 (74)	81 (83)	0.13
Breast drainage	36 (39)	34 (35)	0.5
Axillary drainage	39 (42)	35 (36)	0.3
Compressive bandage	18 (20)	29 (30)	0.1
Breast drainage duration	3 (1–6)	3.5 (1–8)	0.3
Axillary drainage duration (days) med (range)	5 (1–9)	5 (2–7)	0.5

Postoperative course			

All complications	27 (29)	26 (28)	0.8
Hematoma	4 (4)	8 (8)	0.3
Seroma	17 (18)	18 (19)	0.9
*Suture material-relatedcomplications *	12 (13)	8 (8)	0.3
Surgical site infections	10 (11)	6 (6)	0.2
Fever	3 (3)	2 (2)	0.6
Superficial infection	2 (2)	3 (3)	0.7
Deep infection	4 (4)	2 (2)	0.4
Discharge	6 (6)	3 (3)	0.3
Cutaneous complications	7 (8)	3 (3)	0.2
Wound dehiscence	2 (2)	0	0.14
Necroses	0	0	
Wound healing delay	5 (5)	2 (2)	0.2
Allergy	0	1 (1)	0.3
Axillary bridle	1 (1)	0	0.3

**Table 3:** Logistic regression: risk of complications following breast surgery performed on patients of group 1 (traditional suture material).

	OR	*P *
Multifocality	18 (2.2–148)	0.007
Interaction between age and axillary lymphadenectomy	1.04 (1.01–1.07)	0.009

Univariate significant variables: conservative surgery, axillary lymphadenectomy, axillary drainage, breast drainage duration, postoperative anticoagulation, and multifocality.

## Abbreviations

NNIS: National Nosocomial Infection Surveillance
SRC: Suture-related complications
SSI: Surgical site infections
TC: Triclosan-coated.
